# Antisense Phosphorodiamidate Morpholino Oligomers as Novel Antiviral Compounds

**DOI:** 10.3389/fmicb.2018.00750

**Published:** 2018-04-20

**Authors:** Yuchen Nan, Yan-Jin Zhang

**Affiliations:** ^1^Department of Preventive Veterinary Medicine, College of Veterinary Medicine, Northwest A&F University, Yangling, China; ^2^Virginia-Maryland College of Veterinary Medicine and Maryland Pathogen Research Institute, University of Maryland, College Park, MD, United States

**Keywords:** morpholino oligomers, antisense, PMO, PPMO, antiviral compound

## Abstract

Phosphorodiamidate morpholino oligomers (PMO) are short single-stranded DNA analogs that are built upon a backbone of morpholine rings connected by phosphorodiamidate linkages. As uncharged nucleic acid analogs, PMO bind to complementary sequences of target mRNA by Watson–Crick base pairing to block protein translation through steric blockade. PMO interference of viral protein translation operates independently of RNase H. Meanwhile, PMO are resistant to a variety of enzymes present in biologic fluids, a characteristic that makes them highly suitable for *in vivo* applications. Notably, PMO-based therapy for Duchenne muscular dystrophy (DMD) has been approved by the United States Food and Drug Administration which is now a hallmark for PMO-based antisense therapy. In this review, the development history of PMO, delivery methods for improving cellular uptake of neutrally charged PMO molecules, past studies of PMO antagonism against RNA and DNA viruses, PMO target selection, and remaining questions of PMO antiviral strategies are discussed in detail and new insights are provided.

## Introduction

Researchers realized decades ago that antisense nucleic acids could be used to treat diseases. Since then, antisense therapies using a variety of nucleic acids or nucleic acid analogs have been evaluated for use as therapeutic compounds in numerous applications. Among these candidate treatments, morpholino oligos, also known as phosphorodiamidate morpholino oligomers (PMO), have demonstrated promising effectiveness in developmental biology research involving gene knockdown as well as clinical trials focusing on treatments of genetic disorders (Summerton, 2017). Specifically, PMOs are short single-stranded DNA analogs that contain a backbone of morpholine rings connected by phosphorodiamidate linkages (Summerton, 1999). Due to the neutral charged property, they are less likely to interact with proteins while maintain the binding to nucleic acids (Moulton and Jiang, 2009). More specifically, PMO bind to complementary sequences of target mRNA by Watson–Crick base pairing and block mRNA translation through sequence-specific steric blockade. This process is distinct from the RNase H-dependent mechanism for protein translation inhibition, as induced by other antisense compounds such as phosphorothioate DNA (Summerton, 1999). Importantly, PMO are resistant to a variety of enzymes in biologic fluids, which makes them highly suitable for *in vivo* applications (Hudziak et al., 1996).

To date, PMO-based therapy for Duchenne muscular dystrophy (DMD) has shown great success by bypassing effects of a gene mutation underlying a human disease. Specifically, this PMO-based therapy restores production of functional dystrophin by altering RNA splicing to remove the mutated dystrophin exon 51 that disrupts downstream full-length dystrophin protein translation from the mutated mRNA. This drug has been approved by the U.S. Food and Drug Administration and is available in the market under the trade name EXONDYS 51^TM^ (Eteplirsen). Approval of EXONDYS 51^TM^ is a hallmark of PMO-based antisense therapy that demonstrates the potential safety and effectiveness of PMO technology, is driving future development of such therapies for treatment of other diseases. Aside from genetic disorders, PMO therapies have also been evaluated as treatments for other broad categories of untreatable diseases, including viral infections, antibiotic-resistant bacterial infections and cancers (Enterlein et al., 2006; Warfield et al., 2006; Cansizoglu and Toprak, 2017; Chen et al., 2017). In this review, PMO background, as well as research focusing on intracellular delivery of PMO, PMO efficacy against both DNA and RNA viruses, and PMO target selection are outlined. Finally, current challenges for development of successful PMO antiviral strategies are discussed in detail and new insights are provided.

## PMO as a Novel Antisense Strategy

The concept of an antisense oligonucleotide (ON) as a potential therapeutic agent was first demonstrated experimentally through ON targeting of a translation initiation site within Rous sarcoma virus RNA. The ON mechanism was initially explained by its participation in formation of an RNA–DNA duplex with viral RNA that sterically blocks viral gene expression and ultimately prevents viral replication (Zamecnik and Stephenson, 1978). Subsequent studies revealed that ON also participated in a second inhibitory mechanism involving RNA:DNA duplex recognition by the cellular enzyme RNase H, resulting in RNA cleavage and abrogation of virus gene expression as well (Tadokoro and Kanaya, 2009). More recently, RNase H-mediated degradation of target RNA has been shown to be the main RNA-interference mechanism responsible for microRNA (miRNA), small interference RNA (siRNA), and DNA-directed RNA interference (Saurabh et al., 2014). Due to their effectiveness and specificity, such technologies have subsequently attracted the attention of global researchers as tools for therapeutic purposes (Davis et al., 2010; Millington-Ward et al., 2011; Wittrup and Lieberman, 2015).

The first antisense ON tested in clinical trials beginning in 1993 was designed to target p53 for treatment of acute myelogenous leukemia (Bayever et al., 1993). As a medical milestone for the application of antisense technology, the first antisense drug (Fomivirsen) was approved by the FDA in 1998 for treatment of cytomegalovirus retinitis in immunodeficient patients (Marwick, 1998; Wikipedia, 2017). Since then, numerous antisense drugs have been tested in clinical trials for a variety of other human diseases. To date, five antisense compounds have received marketing authorization from the FDA, including Eteplirsen. Currently, more than 100 ongoing clinical trials of antisense compounds are listed on the website ClinicalTrials.gov (Godfrey et al., 2017). However, compared with traditional drug development, industrial development of therapeutic siRNA, DNA-based ON, or ON analogs for control of gene expression have been less successful, as exemplified by the removal of the first commercially available antisense drug, Fomivirsen, from the market.

A key feature underlying the effectiveness of antisense ON is that their nuclease resistance helps them to remain intact for hours in extracellular medium or within cells (Summerton, 1999). Methylphosphonate-linked DNA analogs developed in the late 1970s constituted a major advance in the emerging antisense field, resulting in production of the first antisense drug exhibiting acceptable stability in biological systems (Hudziak et al., 1996). However, in addition to stability, the specificity of RNase-H-based antisense therapy is an important concern, since RNase-H cleaves DNA/RNA duplexes as short as 5- or 6-base pairs in length and is highly active against duplexes that are only 9-10 base pairs in length (Monia et al., 1993;Summerton, 1999). Therefore, RNase-H-independent steric blockage of antisense ON may be a safer strategy. Notably, the effective target region of the steric blocking agent ON for inhibiting translation is generally limited to the 5′-UTR (untranslated region) and start codon regions of mRNA, achieving a good specificity with fewer adverse off-target effects. Furthermore, the binding of an RNase H-independent antisense ON to a partially matched RNA sequence is unlikely to have biological consequences (Lebleu et al., 2008).

**FIGURE 1:**
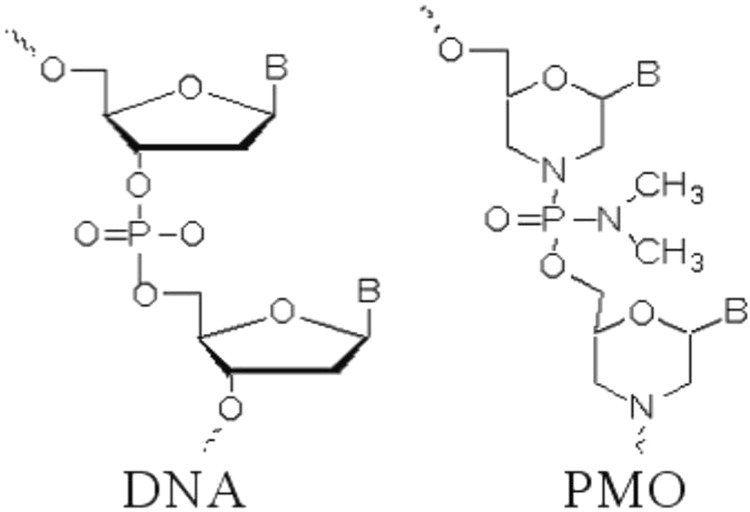
Comparison of chemical structures of PMO and DNA. Illustration of Phosphorodiamidate morpholino oligomer (PMO) contains backbone of morpholine rings connected by phosphorodiamidate linkages. The ribose ring is opened by oxidation, re-closed using ammonia to form a substituted morpholine moiety. The phosphodiester intersubunit bonds are replaced with phosphorodiamidate linkages.

Oligomers possessing a morpholino phosphorodiamidate backbone, also called PMO (**Figure 1**), constitute a novel type of ON analog that is synthesized from ribosides. The ribose ring is opened by oxidation, re-closed using ammonia, which forms a substituted morpholine moiety (Summerton and Weller, 1997). Next, the phosphodiester intersubunit bonds are replaced with phosphorodiamidate linkages (Summerton and Weller, 1997). PMO demonstrate excellent resistance to nucleases, proteases, esterases, and a variety of other enzymes present in biologic fluids (Hudziak et al., 1996). Matrix-assisted laser desorption ionization-time of flight mass spectrometry (MALDI-TOF MS) analysis has demonstrated that PMO are completely resistant to 13 different hydrolases in serum and plasma (Hudziak et al., 1996), bolstering PMO suitability for *in vivo* applications (Hudziak et al., 1996). Furthermore, as uncharged molecules, PMO do not interact strongly with proteins, which minimizes hybridization-independent protein interactions since decreased effectiveness of ON is likely due to their charged phosphorothioate backbone (Moulton and Jiang, 2009; Hagedorn et al., 2017).

**FIGURE 2:**
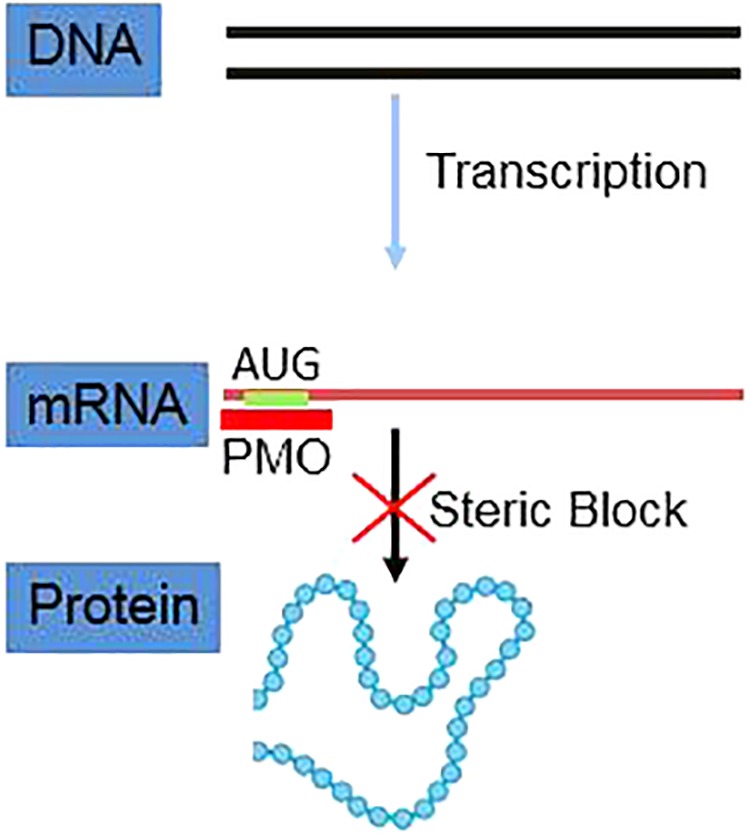
Schematic illustration of PMO inhibiting mRNA translation via steric blockage.

**FIGURE 3:**
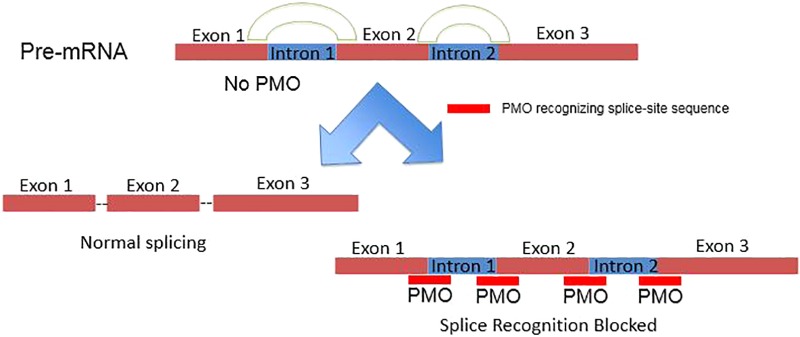
Schematic illustration of PMO inhibiting pre-mRNA splicing via targeting intron–exon junctions.

PMOs bind to complementary sequence in target mRNA by Watson–Crick base pairing to block translation through RNase H-independent steric blockade (**Figure 2**) (Lebleu et al., 2008). Moreover, PMO targeting intron–exon junction sequence is capable to modulate pre-mRNA splicing as well (**Figure 3**) (Havens and Hastings, 2016). For above reasons, antisense PMOs have been a revolutionary tool in developmental biology (Eisen and Smith, 2008). By microinjecting PMOs into eggs or single or pauci-cellular zygotes, PMOs are apportioned into daughter cells during cell division to ensure their delivery to each cell during subsequent cell proliferation (Heasman et al., 2000). In addition to their use for studies of gene function during embryonic development, PMO have been also tested as treatments for a broad range of diseases in numerous clinical trials (mainly promoted by AVI BioPharma, Inc., now Sarepta BioPharma Inc.). However, application of unmodified PMO is greatly limited by inefficient *in vivo* delivery unless a relatively high doses are administrated (Moulton and Jiang, 2009). In fact, in the animal DMD model, high doses of unmodified PMO injection in dystrophic muscle were needed to induce functional dystrophin expression in skeletal muscle (Moulton and Jiang, 2009). Thus, two forms of PMOs with distinct chemical modifications were developed to facilitate intracellular delivery of PMOs, including peptide-conjugated PMO(PPMO) and Vivo-PMO. Moreover, PMOplus, another novel form of positively charged PMO with a morpholino oligomer-based backbone, has been recently developed as the latest version of PMO. These novel PMO forms are discussed in detail below.

## *In Vitro* and *in Vivo* PMO Delivery

Initial techniques to deliver PMO into cultured cells were based on mechanical delivery methods such as microinjection or scraping (Moulton et al., 2003), with associated limitations. More recently, additional mechanical scraping methodologies, electroporation, or use of endosomal escape reagents have been evaluated to improve delivery of ON into cytosolic or nuclear compartments in tissue culture (Partridge et al., 1996; Moulton and Jiang, 2009). Currently, the most intensively developed and widely used *in vivo* PMO delivery strategy is based on the use of arginine-rich cell penetrating peptide (CPP) (Lebleu et al., 2008; Morcos et al., 2008).

Cell penetrating peptide, also known as protein transduction domain (PTD) or Tat-CPP (Dietz and Bahr, 2004), was originally identified after discovery of an unexpected property of the human immunodeficiency virus (HIV) Trans-Activator of Transcription (Tat) protein (Dietz and Bahr, 2004). The novel activity was revealed by observations that Tat could transactivate a HIV-1 LTR promoter after crossing cellular and nuclear membranes (Dietz and Bahr, 2004). Subsequent structural analysis pinpointed a short peptide located in aa 48–60 of Tat (sequence GRKKRRQRRRPPQ) that is responsible for the membrane-crossing activity of the parent Tat protein (Vives et al., 1997; Abes et al., 2008a). This novel peptide sequence, later named as CPP, was then evaluated for its ability to confer membrane-crossing abilities to other proteins and compounds, including PMO. After fluorescence microscopy or flow cytometry analysis of cells treated with fluorescein-tagged PMO, CPP conjugation remarkably enhances cellular uptake of PMO by 8- to 20-fold compared with non-conjugated PMO (Alonso et al., 2005). Moreover, other cationic peptides conjugated PMO were shown to be much less effective than PMO conjugated to Tat-CPP, while Tat-CPP significantly enhanced delivery of PMO to nearly all cells assayed (Moulton et al., 2003). Furthermore, CPP-mediated delivery is a much simpler procedure to conduct than mechanical delivery methods. However, Tat-CPP mediated PMOs delivery required high PMOs concentrations (above 10 μM) to achieve therapeutic antisense activity with cytotoxicity observed. Meanwhile, Tat-CPP PMOs conjugate studies established that the conjugate associated with cell membranes and that internalized conjugate localized to vesicles, cytosol, and nucleus (Moulton et al., 2003). Therefore, CPP sequence optimization, to reduce cytotoxicity and increase uptake efficiency, should enhance PMO effectiveness. This concept has prompted a comparison of two types of CPP (RXR peptide and R_6_Pen peptide) (Lebleu et al., 2008). The most efficient CPP in this study was (R-Ahx-R)_4_R, in which Ahx represents a 6-aminohexanoic acid spacer (Moulton et al., 2007; Lebleu et al., 2008). Importantly, (R-Ahx-R)_4_R-PMO conjugates were shown to be effective in several murine viral infection models (Moulton et al., 2007), as well as in treatment of Duchenne muscular dystrophy. Unfortunately, in some applications, effective doses have approached cytotoxic levels. This obstacle has limited their use in clinical settings (Abes et al., 2008b).

Vivo-PMO exploits a non-peptide-based transporter to deliver PMO into cultured cells or tissues (Morcos et al., 2008). Currently, Gene Tools LLC (Philomath, OR, United States) is the major supplier of Vivo-PMO for research and development applications. Unlike CPP-conjugated PMOs, Vivo-PMO are covalently linked to a molecular scaffold that carries a dendritic structure assembled around a triazine core that holds eight guanidinium head groups optimally oriented for cell membrane penetration (Morcos et al., 2008; Ferguson et al., 2014). Vivo-PMO effectively entered *in vitro* cultured cells as well as a wide variety of mouse tissues *in vivo* to induce correction of a pre-mRNA splicing error, as detected by using an experimental test system designed to detect such an event in cells and tissues (Morcos et al., 2008). Compared with CPP-PMOs, Vivo-PMO have been less frequently investigated for inhibitory effects against target genes. However, available data suggest that at least a 50% knockdown of target genes can be achieved using Vivo-PMO, with no adverse side effects both *in vitro* and *in vivo* (Guo et al., 2011; Kang et al., 2011; Nazmi et al., 2012; Reissner et al., 2012). Meanwhile, mouse model studies have also shown that intravenous (IV) and intraperitoneal (IP) administration of Vivo-PMO were equally efficacious (Reissner et al., 2012; Sartor and Aston-Jones, 2012). Furthermore, it appears that Vivo-PMO is less cytotoxic than CPP-PMO, since only one report has demonstrated cytotoxicity of Vivo-PMO (Ferguson et al., 2014). It appears that the dendrimer of Vivo-PMO is capable to induce red blood cell sedimentation, prompting Ferguson et al. to recommend that oligonucleotide analogs should be analyzed for potential 3′ to 5′ base pair hybridization that may induce dendrimer clustering. Moreover, supplementation of Vivo-PMO with physiological saline or anticoagulation therapy holds promise for counteracting Vivo-PMO toxicity (Ferguson et al., 2014).

**FIGURE 4:**
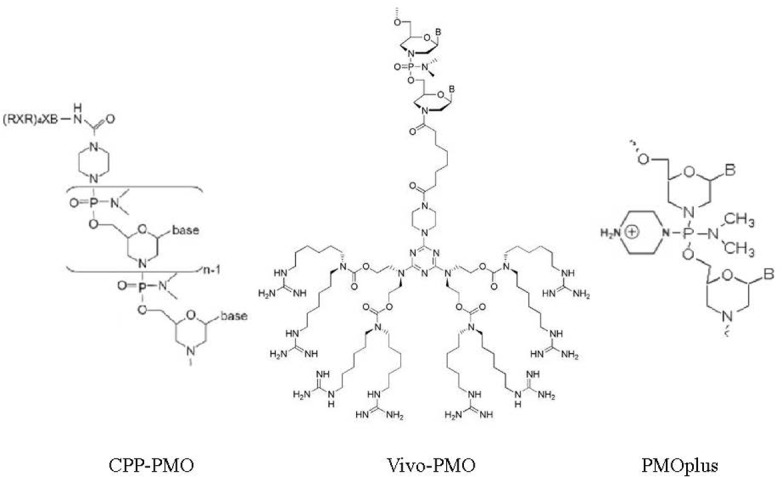
Chemical structures of CPP-PMO, Vivo-PMO, and PMOplus^TM^. PMO conjugated with cell penetrated peptide (R-Ahx-R)_4_R, in which Ahx represents a 6-aminohexanoic acid spacer. Vivo-PMO are covalently linked to a molecular scaffold that carries a dendritic structure assembled around a triazine core that holds eight guanidinium head groups optimally oriented for cell membrane penetration. PMOplus^TM^ is charged PMO that contains positively charged piperazine groups within its molecular backbone.

Compared to CPP-conjugated PMO and Vivo-PMO, PMOplus^TM^ is newer type of charged PMO that contain positively charged piperazine groups within its molecular backbone (Warren et al., 2012). PMOplus is the most recently developed form of PMO and studies have demonstrated this type of PMO is well tolerated and exhibits improved efficacy in numerous in *in vivo* viral infection models relative to other PMO therapies (Swenson et al., 2009; Warren et al., 2010, 2015, 2016; Meng et al., 2017). However, PMOplus is still unavailable to most researchers due to its proprietary status as technology solely owned by AVI. However, there are reports available to date suggest that PMOplus may be less cytotoxic than CPP-PMO. In a phase I clinical trial to evaluate PMOplus compounds against Ebola virus, 30 healthy male and female subjects between 18 and 50 years of age in six dose-expansion cohorts of 5 subjects per dosage group, each received a single i.v. infusion of the active study drug (0.005, 0.05, 0.5, 1.5, 3, or 4.5 mg/kg PMOplus). Results demonstrated that PMOplus treatments were safe and well tolerated at these doses studied (Heald et al., 2014), with safety superior to that of CPP-PMO or Vivo-PMO. However, a systematic investigation is still needed to compare Vivo-PMO and PMOplus with regard to efficiency, stability, and cytotoxicity. An illustration of chemical structures of CPP-PMO, Vivo-PMO and PMOplus^TM^ was listed as **Figure 4**.

## Evaluation of PMO as Antiviral Compounds Against RNA Viruses

PMO have been explored as antiviral compounds against RNA viruses, including Ebola virus, flavivirus, coronavirus, picornavirus, and others. In this section, major advances in this research field are presented.

### PMO Designed Against Ebola Virus

The filoviruses, including Marburg virus and Ebola virus (EBOV), are negative-sense, single-stranded RNA viruses that are highly pathogenic, causing human outbreaks of viral hemorrhagic fever with up to 90% fatality. The 2013–2016 epidemic of Ebola virus disease caused 11,323 deaths of 28,646 total cases in West Africa (Fischer et al., 2017). Because currently no commercial vaccines or effective therapeutics are yet available for filovirus infections (Fischer et al., 2017; Reynolds and Marzi, 2017; Trad et al., 2017), antiviral drugs are urgently needed. Thus, both peptide-conjugated PMO (PPMO) and non-conjugated PMOs have been tested against Ebola virus infection in cultured cells and animal models. An earlier study showed that a 22-mer PPMO targeting the translation start site region of EBOV VP35 positive-sense RNA exhibited sequence-specific, time- and dose-dependent inhibition of EBOV replication in cultured cells (Enterlein et al., 2006). Moreover, this PPMO provided complete protection of mice when administered before or after challenge with a lethal dose of EBOV. Interestingly, a corresponding non-conjugated PMO also provided protection of mice when administered prophylactically as well. Meanwhile, another report in same year demonstrated that a combination of EBOV-specific PMOs targeting viral mRNAs for VP24, VP35, and RNA polymerase L protected rodents against EBOV challenge when administered before and after exposure (Warfield et al., 2006). In the same study, non-conjugated PMO were also tested in a prophylactic proof-of-principal trial in rhesus macaques whereby the same PMO formulation protected 75% of macaques from lethal EBOV infection.

More recently, PMOplus has also been shown to be effective against Ebola infection in monkeys. When delivered 30–60 min after infection, PMOplus AVI-6002 (composed of AVI-7357 and AVI-7539 that target EBOV VP24 and VP35, respectively) (Iversen et al., 2012) protected over 60% of rhesus monkeys against lethal infection with Zaire Ebola virus (ZEBOV) (Warren et al., 2010). Similarly, PMOplus AVI-6003 (composed of AVI-7287 and AVI-7288 that target VP24 and VP35 of Marburg virus, respectively) protected 100% of cynomolgus monkeys against infection with Lake Victoria Marburg virus (MARV) when delivered after infection (Iversen et al., 2012). Therefore, PMOplus holds great promise for treatment of patients infected with these highly pathogenic viruses. In another study, the same research group showed that PMOplus AVI-7537 targeting VP24 alone was sufficient to protect monkeys against lethal EBOV infection, whereas PMOplus AVI-7539, targeting VP35 alone, failed to do so (Warren et al., 2015). These results thus confirm that VP24 may be a key EBOV virulence factor and may serve as a promising target for further development of effective anti-EBOV treatment strategies.

### PMO Against Picornaviruses

Picornaviruses are non-enveloped positive-sense, single-stranded RNA viruses belong to the family *Picornaviridae*. This family includes more than 30 genera and 75 species (Zell et al., 2017), with RNA genomes length ranging from 6.7 to 10.1 kb. Many picornaviruses are important human and animal pathogens, including poliovirus, coxsackievirus B3 (CVB3), enterovirus 71 (EV-71), and foot-and-mouth disease virus (FMDV). No vaccine yet exists for picornaviruses other than for poliovirus and FMDV. Moreover, no effective antiviral therapy yet exists for treatment of infections caused by any pathogenic picornavirus. However, PPMO targeting conserved internal ribosome entry site (IRES) sequences have been shown to be highly effective in protecting cultured cells against infection by human rhinovirus type 14, coxsackievirus type B2, and poliovirus type 1 (PV1) (Stone et al., 2008), with reduction of PV1 titers by up to 6 log10. This 22-mer PPMO (EnteroX) targets an IRES sequence that is identical for >99% of all human enteroviruses and rhinoviruses which has been successfully used to treat poliovirus receptor (PVR) transgenic mice to prevent PV1 infection after challenge with three times the 50% lethal dose(LD_50_). This result also showed that mice receiving PPMO treatment exhibited an approximately 80% higher survival rate than controls, with significant reduction of viral titers in small intestine, spinal cord, and brain (Stone et al., 2008).

Coxsackievirus B3 (CVB3) is a primary cause of viral myocarditis, without any effective therapy. In study tested eight CVB3-specific PPMO in cultured HeLa cells and HL-1 cardiomyocytes, as well as in a murine infection model (Yuan et al., 2006). Among eight PPMOs tested, PPMO-6, designed to target the 3′ portion of the CVB3 IRES, was especially potent against CVB3 replication in cultured cells. When cells were treated prior to or shortly after CVB3 infection, virus proliferation was significantly inhibited, with approximate 3 log10 decrease of viral titers. In A/J mice, PPMO-6 intravenous administration once prior to and once after CVB3 infection significantly reduced cardiac tissue damage, with notable decreases in myocardium virus titers than control (Yuan et al., 2006).

Enterovirus 71 (EV-71) generally causes mild hand-foot-and-mouth disease, but severe neurological complications with high mortality rates have been reported. In one study, testing of Vivo-PMO designed to target the EV-71 IRES and the RNA-dependent RNA polymerase (RdRp) was performed (Tan et al., 2014). Vivo-PMO targeting EV-71 IRES significantly reduced EV-71 replication in human embryonal rhabdomyosarcoma RD cells. In contrast, Vivo-PMO targeting EV-71 RdRp was less effective. The results suggest that IRES-targeting Vivo-PMO are potential antiviral candidates that can abrogate early EV-71 infection (Tan et al., 2014).

FMDV causes a highly contagious viral disease of cloven-hoofed animals that can lead to severe economic losses to the livestock industry. Six PPMOs were designed to target 5′ and 3′ UTRs of the FMDV genome (strain A(24) Cruzeiro/Brazil/1955 [A(24)Cru]) and were evaluated in cultured cells (Vagnozzi et al., 2007). Three of the PPMOs, targeting domains including the 5′ portion of the IRES and the two translational start codon-containing regions, were highly effective in inhibiting FMDV replication. At low micromolar concentrations, PPMO led to a dose-dependent and sequence-specific virus titer reduction of over 5 log10, while three other PPMO that targeted other genome regions were less effective (Vagnozzi et al., 2007).

### PMO Against Members of Nidovirales

The order *Nidovirales* includes the families *Coronaviridae*, *Arteriviridae*, *Roniviridae*, and *Mesoniviridae* (Cong et al., 2017). The members of this group are positive-sense, single-stranded RNA viruses. The *Coronaviridae* and *Arteriviridae* include groups of viruses infecting vertebrates (mainly mammalian species), whereas the other two families include viruses infecting invertebrates. PMO have been tested as antivirals against members of both *Coronaviridae* and *Arteriviridae.*

Members of *Coronaviridae* include severe acute respiratory syndrome coronavirus (SARS-CoV) and Middle East respiratory syndrome coronavirus (MERS-CoV), which are recently identified as potent human pathogens (Cong et al., 2017). Mouse hepatitis virus (MHV), also belongs to this family, which has long served as a model for understanding viral hepatitis in humans and to assess PMO antiviral compounds (Neuman et al., 2004; Burrer et al., 2007). PPMO targeting the MHV replicase exhibited low toxicity in DBT astrocytoma cells, a cell line for studying MHV infection (Neuman et al., 2004). A later study tested PPMOs against several MHV strains in cell culture and *in vivo* using mouse models (Burrer et al., 2007; Moulton et al., 2007). Among ten PPMOs against various viral genome target sites, PPMO 5TERM, which targeted the 5′ terminus of the RNA genome, was found to be highly effective in inhibiting six different MHV strains. In mice, 5TERM PPMO treatment led to prevention of virus-induced tissue damage. Prophylactic treatment with 5TERM PPMO also decreased MHV-induced weight loss and prolonged post-challenge survival. This study also showed that no weight loss or detectable histopathologic changes were observed after prolonged PPMO treatment of uninfected mice (Burrer et al., 2007). Besides, PPMO have also been shown to inhibit SARS-CoV replication as well (Neuman et al., 2005). Among all PPMO tested, two PPMO targeting the viral transcription-regulatory sequence (TRS) within the 5′ UTR brought about the most significant inhibition of CPE and reduced cell-to-cell spread when administered prior to peak viral proliferation in cultured cells (Neuman et al., 2005).

Members of the *Arteriviridae* include an economically important virus, porcine reproductive and respiratory syndrome virus (PRRSV). PRRSV causes a contagious swine disease characterized by reproductive failure in sows and respiratory disease in pigs of all ages. The disease has plagued the global swine industry since it was first reported in 1987 and current strategies used for PRRS control are still inadequate (Du et al., 2017). Research in our laboratory has focused on development of PPMOs against PRRSV. Based on the genome sequence and viral protein function, a series of PPMOs with various targets were designed that included three PPMO targeting the 5′ end UTR of the PRRSV genome, namely 5UP1, 5UP2, and 5HP, as well as six PPMO targeting the translation initiation regions of ORFs 2-7, namely 2P1, 3P1, 4P1, 5P1, 6P1, and 7P1 (Patel et al., 2008; Han et al., 2009). PPMO targeting the 5′ UTR were highly effective for inhibiting PRRSV replication in cell culture in a dose-dependent and sequence-specific manner. Specifically, PPMO 5UP2 or 5HP caused a 4.5 log10 reduction of PRRSV yield. Moreover, 5UP2 and 5HP also demonstrated broad inhibition of heterogeneous PRRSV isolates (Patel et al., 2008). In addition, if PPMO 5UP1 targeting the 5′ UTR of PRRSV genome was paired with PPMO 4P1 and 7P1, which targeted translation initiation regions of ORFs 4 and 7, respectively, the combination enhanced inhibition of heterologous strains of the North American PRRSV genotype more effectively than individually *in vitro* (Han et al., 2009). Inhibition was verified at both PRRSV RNA and protein expression levels.

Since PPMO 5UP2, which targets a highly conserved sequence within the 5′-terminal region of PRRSV genome, effectively induces a multi-log10 inhibition of PRRSV replication *in vitro*, we also conducted an *in vivo* evaluation of this PPMO (Opriessnig et al., 2011). PRRSV-negative 3-week-old piglets received PPMO intranasally at 24 h before infection as well as 2 and 24 h after PRRSV infection. PPMO treatment was well tolerated in piglets, with no weight change observed across all piglet groups and PPMO administration significantly reduced PRRSV viremia and interstitial pneumonia. Moreover, in alveolar macrophages isolated at 14 days post-infection, elevated expression of antiviral genes in PPMO-treated piglets was observed as well (Opriessnig et al., 2011).

Besides PRRSV, antiviral effects of PMO were also evaluated against equine arteritis virus (EAV), another member of the family *Arteriviridae* (van den Born et al., 2005). Similar to PRRSV studies above, PPMO designed to target the 5′ terminal UTR of the EAV genome remarkably reduced virus replication in a sequence-specific and dose-responsive manner. However, PPMO that targeted 3′-terminal regions of the viral genome or its anti-genome only resulted in moderate reduction of EAV replication when relatively high concentrations of the PPMOs were applied. Moreover, PPMO targeting the EAV TRS, which is essential for subgenomic RNA synthesis, were ineffective to achieve transcription interference (van den Born et al., 2005). However, PPMO targeting the 5′ UTR of EAV were able to cure viral infection of persistently infected HeLa cells (Zhang et al., 2010).

### PMO Designed Against Viruses of the Genus *Flavivirus*

*Flavivirus* is a viral genus within the family *Flaviviridae*. This genus includes West Nile virus (WNV), dengue virus (DENV), yellow fever virus, Zika virus, and several other viruses which may cause encephalitis (Coffey et al., 2014; Musso and Gubler, 2016). Except for yellow fever virus, no effective vaccine or antiviral drug exists against flaviviruses, prompting evaluation of PMO against several members of *Flavivirus*. Among a panel of PPMO against WNV, PPMO targeting the 5′- and 3′-termini of the WNV genome, designated 5′End or 3′CSI, exhibited the greatest potency in blocking WNV replication (Deas et al., 2005). Moreover, treatment of WNV-infected cells with either 5′End or 3′CSI PMO led to a significant reduction of virus titers by approximately 5–6 log10 without apparent cytotoxicity. PPMO 5′End inhibited WNV translation, whereas PPMO 3′CSI suppressed WNV RNA replication. Meanwhile, PPMO 3′CSI also inhibited other mosquito-borne flaviviruses when the targeted 3′CSI-like sequences which were relatively conserved to the respective WNV 3′CSI target sequence. Therefore, PPMO targeting conserved *cis*-acting elements of flavivirus genomes should be explored as anti-flavivirus therapeutics (Deas et al., 2005). More recently, both PPMO 5′End or 3′CSI were also tested in a mouse model of WNV infection and provided partial protection when administered at 100 or 200 μg/day. Moreover, minimal to no PPMO-mediated toxicity observed, while toxicity was observed at a larger dosage of 300 μg/day (Deas et al., 2007).

A panel of PPMO was also tested against DENV (Kinney et al., 2005) whereby PPMO targeting 3′-terminal nucleotides of the serotype 2 DENV (DEN-2) virus genome exhibited relatively poor suppression of DEN-2 virus titer. However, moderate reduction of titer was observed for PPMO targeting either the AUG translation start site region of the single open reading frame or the 5′ cyclization sequence region. The most highly effective PPMO were 5′SL and 3′CS (targeting the 5′-terminal nucleotides and the 3′ cyclization sequence region, respectively), which reduced viral titer by greater than 5.7 log10 compared to controls at 6 days post-infection with DEN-2 virus. Notably, treatment with 10 μM 3′CS inhibited replication of all four DEN virus serotypes by over 4 log10, in most cases to below detectable limits (Kinney et al., 2005).

A third PPMO that was designed to target the top of the 3′ stem-loop (3′SLT) inhibited DEN replication in BHK cells (Holden et al., 2006). The inhibitory mechanism was studied using a novel DEN2 reporter replicon and a DEN2 reporter mRNA. The results demonstrated that 5′SL inhibited viral translation and 3′CS blocked viral RNA synthesis but not viral translation, whereas the 3′SLT inhibited both viral translation and RNA synthesis (Holden et al., 2006). More recently, anti-DENV PPMO 5′SL and 3′CS were also tested in AG129 mice before and/or after infection with DENV-2 (Stein et al., 2008). Intraperitoneal (ip) infection of AG129 mice with 10^4^–10^6^ pfu of DENV-2 (strain New Guinea C) shortened survival to 9–17 days. When 5′SL or 3′CS were administered before and after DENV infection, the average survival time was extended by up to 8 more days. This study also included pharmacokinetic and toxicology analysis of non-infected animals. The results showed that the mice had high concentrations of PPMO in liver following nine consecutive once-daily ip treatments of 10 mg/kg PPMO, with little impact on overall mouse health (Stein et al., 2008).

PMO and PPMO have also been studied for their ability to prevent or treat Japanese encephalitis virus (JEV) infection as well. A PPMO (P10882) targeting the 3′ cyclization sequence (3′CSI) of JEV exhibited significant antiviral activity in Vero (epithelial), Neuro2A (neuronal), and J774E (macrophage) cells at non-toxic concentrations (Anantpadma et al., 2010). Addition of P10882 to cells before infection decreased JEV replication to undetectable levels in Vero cells and resulted in a 93 and 66% reduction in JEV titer in J774E and Neuro2A cells, respectively. In this study, antiviral effects of P10882 were also assessed *in vivo*. When treated intracerebrally with a 20 mg/kg dose of P10882 every 12 h for 5 days, 60–80% of 1-week-old mice were protected from a lethal dose of JEV (Anantpadma et al., 2010). Meanwhile, testing of Vivo-PMO targeting the 5′ and 3′ UTR of the JEV genome have also been conducted in mice (Nazmi et al., 2010). Administration of intraperitoneal injections of Vivo-PMO (5 mg/kg body weight) daily for up to 5 days immediately after JEV infection of mice prolonged survival, with reduced viral load and viral protein expression in brain. Moreover, proinflammatory cytokine levels in brain, which normally increase after JEV infection, were reduced following PMO treatment and align with observations of reduced microglial activation in brain as well (Nazmi et al., 2010).

Chikungunya virus (CHIKV) causes infection in humans that is associated with debilitating and persistent arthralgia and arthritis. Two PPMO were designed to target highly conserved sequences present in CHIKV non-structural and structural polyproteins (Lam et al., 2015). CPMO1, a PPMO that targets the ORF1 AUG region, significantly suppressed CHIKV replication in HeLa cells when administered before infection. Notably, in neonatal mice, administration of 15 μg/g CPMO1 before infection conferred 100% survival against CHIKV disease (Lam et al., 2015).

### PMO Designed Against Negative-Strand RNA Viruses

PPMO have also been tested for antiviral effects toward group V (-)ssRNA virus families that include *Pneumoviridae*, *Paramyxoviridae*, *Orthomyxoviridae*, *Arenaviridae*, and others. Respiratory syncytial virus (RSV), a member of the family *Pneumoviridae*, is a major cause of lower respiratory tract infections in infants, young children, and high-risk adults. Currently, no vaccine exists to prevent RSV infection. However, two antisense PPMOs designed to target the 5′-terminal region and the translational start site of RSV L mRNA have been tested and exhibited minimal cytotoxicity (Lai et al., 2008). PPMO AUG-2 inhibited RSV replication by reducing viral titers by over 2 log10. When administered before RSV intranasal inoculation, PPMO AUG-2 protected BALB/c mice from infection, with reduced viral titers in lung tissue and attenuation of pulmonary inflammation (Lai et al., 2008).

Measles virus (MeV) is a member of the family *Paramyxoviridae*. MeV is a highly contagious human pathogen that can be treated effectively with available antiviral compounds and prevented using a vaccine. Five PPMOs targeting MeV genomic RNA or mRNA were tested in cultured cells (Sleeman et al., 2009). PPMO 454, targeting a conserved sequence in the translation start site of the mRNA coding for viral nucleocapsid protein, was highly effective against multiple genotypes of MeV (Sleeman et al., 2009).

Influenza A virus, a member of the family *Orthomyxoviridae*, is a relentless ongoing global public health concern. When delivered by intranasal administration, PPMO inhibited replication of equine influenza A virus FLUAV A/Eq/Miami/1/63 (H3N8) in mice and exhibited no toxicity at effective antiviral concentrations *in vivo* (Lupfer et al., 2008). Meanwhile, a PPMO panel was developed to target RNA genome segments encoding polymerase subunits of a highly pathogenic mouse-adapted influenza A virus strain (SC35M; H7N7) (Gabriel et al., 2008). In this study, virus replication in MDCK cells was significantly inhibited by three PPMO targeting either the translation start site region of PB1 or NP mRNA or the 3′-terminal region of NP viral RNA (vRNA). In a mouse model, when PPMO targeting the PB1-AUG region or NP vRNA were administered intranasally once 3 h before and once 2 days after intranasal infection with a lethal dose of SC35M, treated mice exhibited significantly lower viral titers in lungs and 50% greater survival versus untreated controls over the 16-day duration of the experiment (Gabriel et al., 2008).

Besides targeting viruses, PPMO have also been tested for efficacy against host mRNA encoding proteases crucial for viral infectivity. Such PPMO can block host protease cleavage of the influenza virus hemagglutinin (HA) to inhibit viral infectivity. Treatment of human Calu-3 airway epithelial cells with a PPMO T-ex5, designed to interfere with splicing of HA-cleaving protease TMPRSS2 pre-mRNA, resulted in production of TMPRSS2 mRNA lacking exon 5 that resulted in production of an enzymatically inactive form of TMPRSS2 (Bottcher-Friebertshauser et al., 2011). Ultimately, T-ex5 PPMO was shown to prevent cleavage of HAs of various human seasonal and pandemic influenza A viruses, leading to significant reduction of viral titers by 2 to 3 log10 (Bottcher-Friebertshauser et al., 2011).

Junín virus, a threat to human health and a member of the *Arenaviridae* family, can cause meningitis and hemorrhagic fever. PPMO designed to interfere with translation have been shown to be effective in reducing Junín virus replication (Neuman et al., 2011). In cultured cells, PPMO target sequences located at the 5′ termini of both genomic segments are highly conserved across the arenaviruses. Consequently, these PPMO are effective against Junín virus, Tacaribe virus, Pichinde virus, and also against lymphocytic choriomeningitis virus (LCMV) whereby they suppress viral titers in livers of LCMV-infected mice (Neuman et al., 2011).

### PMO Against Alphaviruses or Other Alphavirus-Like Positive-Sense RNA Viruses

The genus *Alphavirus* includes positive-sense RNA viruses that threaten human health (Paessler et al., 2008). Sindbis virus (SINV) is a member of the family *Togaviridae*. PPMO targeting both the 5′-terminus and AUG translation start site of the SINV genome significantly suppressed SINV replication in tissue culture (Paessler et al., 2008). Venezuelan equine encephalitis virus (VEEV) is another member of the *Togaviridae.* PPMO targeting VEEV regions corresponding to SINV regions mentioned above inhibit several strains of VEEV *in vitro*. Notably, mice pre-treated with PMO were protected from lethal VEEV infection, while only partial protection was observed for mice receiving only post-infection PMO treatment (Paessler et al., 2008).

Noroviruses, which belong to the *Caliciviridae* family, are non-enveloped, positive-sense, single-stranded RNA viruses with genomes of approximately 7.5 kb in length that encode three ORFs. Noroviruses cause non-bacterial epidemic gastroenteritis (Bok et al., 2008). PPMO targeting the first AUG region of the ORF1 near the 5′-end of the murine norovirus (MNV) genome effectively inhibited MNV replication in cultured cells (Bok et al., 2008). Moreover, a consensus PPMO targeting the corresponding 5′-end of the genome of several diverse human norovirus genotypes also inhibited Norwalk virus protein expression (a species of *Norovirus*) in replicon-bearing cells in a cell-free luciferase reporter assay (Bok et al., 2008).

Similar to noroviruses, hepatitis E virus (HEV) is a positive-sense, single-stranded RNA virus containing three ORFs and is currently classified within the family *Hepeviridae* (Nan and Zhang, 2016). HEV shares a similar genome structure with members of *Caliciviridae* and was previously classified in that family (Nan and Zhang, 2016). Our study demonstrated that PPMO targeting the 5′ terminal of the HEV genome at the start codon of ORF1 are highly effective against both HEV genotypes 1 and 3 infection *in vitro* (Nan et al., 2015). Moreover, PPMO targeting the 3′ UTR of the HEV genome or the 5′ terminus of antisense HEV RNA can also block HEV replication, but to a lesser extent than achieved by PPMO targeting the 5′ terminus (Nan et al., 2015). Since the 3′ UTR and 5′ terminus of antisense HEV RNA are generally considered binding sites for HEV RNA-dependent RNA polymerase (RdRp), it appears that PPMO-mediated steric blockade also applies to RdRp as well (Nan et al., 2015).

## Evaluation of PMO as Antiviral Compounds Against DNA Viruses

As compared with PMOs’ applications against RNA viruses, PMOs use against DNA viruses has been much less studied. To date, only members of *Herpesviridae* have been tested for PMO-mediated inhibition. Research from our lab evaluated PMOs against Kaposi’s sarcoma-associated herpesvirus (KSHV). KSHV, also known as human herpesvirus 8 (HHV-8), is a human oncovirus belonging to the gamma herpesvirus subfamily. KSHV is associated with Kaposi’s sarcoma (KS) and two B-cell lymphoproliferative diseases: primary effusion lymphoma (PEL) and multicentric Castleman’s disease (MCD) (Purushothaman et al., 2016). These diseases are AIDS-related malignancies in HIV-infected patients. KSHV infection in humans exhibits either a lifelong immunologically silent and latent infection or a transient lytic infection with distinct viral gene-expression profiles (Lagunoff et al., 2002; Bechtel et al., 2003). During the predominantly latent state of KSHV infection, the KSHV genome is maintained as circular, extra-chromosomal DNA that replicates inside host cells in a cell cycle-dependent manner. Expression of a few key viral regulators, such as latency-associated nuclear antigen (LANA) encoded by ORF73, viral cyclin (vCyclin) encoded by ORF72, and viral FLIP (vFLIP) encoded by ORF71, are observed during latency (Uppal et al., 2014). Upon reactivation to assume a lytic infection state, a full repertoire of lytic viral genes, including ORF50 (transcription activator, RTA), ORF57, ORF59, ORF40, ORF6, ORF9, ORF-K8, ORF-K9 (vIRF-1), ORF-K2 (viral interleukin-6, vIL-6), ORF74 (viral G protein-coupled receptor vGPCR), and viral chemokines (ORF-K6/vCCL-I and ORF-K4/vCCL-II) are expressed in a temporally-regulated manner (Purushothaman et al., 2016).

Based on the functions of KSHV latent and lytic genes, a panel of PPMOs was designed against a set of genes including LANA, vIL-6, RTA, and vIRF-1 (Zhang et al., 2007, 2008, 2011). Treatment of KSHV-positive PEL cells with an RTA-specific PPMO RP1 not only reduced RTA expression but also caused down-regulation of several other early and late KSHV gene products, including vIL-6, vIRF-1, and ORF-K8.1A. Moreover, KSHV DNA copy numbers both in PPMO RP1-treated PEL cells and culture supernatants were reduced, demonstrating inhibition of KSHV lytic replication (Zhang et al., 2007). Furthermore, treatment of BCBL-1 cells with PPMO against LANA reduced LANA expression (Zhang et al., 2007). Meanwhile, PPMO against vIL-6 and vIRF-1 were also evaluated. The viral homologue of the proinflammatory cytokine IL-6, vIL-6, is believed to contribute to vascular permeability and formation of PEL effusions (Sakakibara and Tosato, 2011). This cytokine shares low but significant homology to human IRF family members and acts as an oncogene to inhibit interferon induction (Gao et al., 1997). Treatment of PEL cells with PPMO designed against vIL-6 mRNA led to marked reduction in the proportion of vIL-6-positive PEL cells and reduced both the growth of PEL cells and KSHV DNA levels (Zhang et al., 2008).

Meanwhile, PPMO targeting vIRF-1 have also been shown to inhibit viral DNA replication in addition to blocking vIRF-1 expression in BCBL-1 cells (Zhang et al., 2011). Interestingly, reduction of vIRF-1 expression in KSHV-infected cells resulted in higher expression levels of cellular IRF-3 and of the signal transducer and activator of transcription 1 (STAT1) (Zhang et al., 2011). Encouraged by these *in vitro* data, an *in vivo* evaluation of PPMO effects on expression of multiple viral genes was conducted (Zhang et al., 2011). However, *in vivo* results defied expectations drawn from *in vitro* studies. Specifically, only PPMO against vIL-6 demonstrated promising *in vivo* inhibition whereby SCID mice treated with this PPMO exhibited no engraftment of KSHV-infected PEL cells and remained healthy throughout the 120-day study (Zhang et al., 2011). Conversely, in SCID mice receiving a combination of PMO against two vIRF-1, there was a trend of less engraftment of KSHV-infected PEL cells, but the difference in results was not statistically significant between treated and control mice. Therefore, PPMO targets selected using *in vitro* assessments may not achieve expected inhibition *in vivo*, emphasizing the fact that careful validation using adequate animal models is necessary.

Acyclovir (ACV) is a nucleic acid analog of guanosine that is used to treat HSV. Viral resistance to ACV has become a recent concern. As a potential treatment for ACV-resistant HSV, PPMO against resistant HSV-1 and HSV2 were evaluated both *in vitro* and in a mouse model as well. For HSV-1, ICP0 and ICP27 were selected as virus PPMO targets. ICP0 from both HSV-1 and HSV-2 acts as an E3 ubiquitin ligase to antagonize host innate immunity (Halford et al., 2011; Lanfranca et al., 2014). ICP27 is a multiple function regulator involved in pre-mRNA splicing and the host innate immune response (Christensen et al., 2016; Tang et al., 2016). When PPMO targeting translation start site regions of HSV-1 ICP0 or ICP27 mRNA were applied before or soon after HSV-1 infection of cultured cells, a 70–98% reduction of HSV-1 yield was observed, as assessed by reduced plaque formation. Moreover, ICP0-specific PPMO also inhibited ACV-resistant HSV-1 plaque formation by 70–90%, while an equivalent dose of ACV only led to a 40–50% plaque reduction (Moerdyk-Schauwecker et al., 2009).

*In vivo* data suggest that PPMO are well-tolerated in uninfected mice after 7 days of administration of 100 μg/day (Moerdyk-Schauwecker et al., 2009). Topical application of 10 μg ICP0-specific PPMO into the eyes of HSV-1-infected mice reduced the incidence of eye disease by 37.5–50% compared to controls. Therefore, PPMO holds promise as an antiviral drug for use in treating HSV-1 ocular infection (Moerdyk-Schauwecker et al., 2009). With regard to HSV-2, PPMO targeting ICP0 or ICP27 mRNA were also highly effective against either non-ACV-resistant or ACV-resistant HSV-2 strains. In one *in vivo* study, PPMO were well-tolerated in BALB/c mice and cotton rats. Cotton rats receiving ICP27-specific PPMO 24 h after HSV-2 inoculation showed a reduction in genital lesions and a 37.5% reduction in mortality at 14 days post-infection. Mice receiving a combined regimen of 100 μM of ICP27- and ICP0-specific PPMO before HSV-2 inoculation were completely free from genital viral infection at 3–5 days post-inoculation (Eide et al., 2010).

## Target Selection and Potential for Emergence of Mutants Resistant to Antisense PMO

A variety of studies suggest that PMO would be good candidate for antiviral therapeutics against emerging or reemerging viruses in the absence of other effective therapies. However, effective target sequences for PMO design need to be carefully validated. A list of previously published effective virus target regions used for PMO designed against RNA viruses are briefly summarized in **Table 1**. To date, for most PMOs evaluated against positive-sense RNA viruses, targeting sequences of PMO are mainly located in the 5′ terminal ends of viral genomes (**Table 1**). Notably, it appears mRNA-like properties of positive-sense RNA virus genomes make them highly susceptible to PMO-mediated translation inhibition if the PMOs pairing sequence are located at the 5′ terminal end of the viral genomes, with few exceptions. Moreover, UTR at either end of viral genomes are generally conserved among viral strains. Conversely, for some positive-sense RNA viruses, PMO targeting of the 3’ terminal ends of the viral genome were tested as well. However, data appears to be mixed for 3′ terminal-targeting PMOs. On the one hand, for members of *Flaviviridae*, PMOs targeting the 3’ cyclization sequence located within the 3′ terminal end are highly effective in inhibiting virus replication (Deas et al., 2005, 2007; Kinney et al., 2005; Holden et al., 2006; Stein et al., 2008; Anantpadma et al., 2010; Nazmi et al., 2010). On the other hand, the use of PMO targeting either the 3′-terminal regions of the viral genome or of the negative genomic strand only resulted in moderate reduction of EAV (van den Born et al., 2005). This result suggests that PMO targeting the 3′ terminal end are less effective for inhibiting EAV replication compared to PMO targeting the 5′ terminal UTR of the EAV genome. Therefore, the viral genome 5′ end is the preferred target region for antiviral PMO design against positive-sense RNA viruses.

**Table 1:** Targeting regions of PMOs that are effective against RNA viruses.

Polarity of RNA genome	Virus order/family	Virus	PMO targeting region	Reference
Positive-sense RNA virus	*Picornavirales*	Coxsackievirus B2 and B3,	IRES in 5′ terminal end of genome	Yuan et al., 2006; Stone et al., 2008
		Poliovirus type 1	IRES in 5′ terminal end of genome	Stone et al., 2008
		Enterovirus 71	IRES in 5′ terminal end of genome, RNA-dependent RNA polymerase	Tan et al., 2014
		Foot-and-mouth disease virus	5′ portion of the IRES, Translation initiation codon region	Vagnozzi et al., 2007
	*Nidovirales*	Mouse hepatitis virus	5′ terminus of the genomic RNA	Burrer et al., 2007
		SARS-Coronavirus	Transcription-regulatory sequence (TRS) in the 5′ UTR	Neuman et al., 2005
		Porcine reproductive and respiratory syndrome virus (PRRSV)	5′ UTR of the PRRSV genome	Patel et al., 2008; Han et al., 2009; Opriessnig et al., 2011
		Equine arteritis virus (EAV)	5′ UTR of EAV genome	van den Born et al., 2005; Zhang et al., 2010
	*Flaviviridae*	West Nile virus	5′ and 3′ termini of genome	Deas et al., 2005, 2007
		Dengue virus	5′ and 3′ termini of the genome, 3′ stem-loop	Kinney et al., 2005; Holden et al., 2006; Stein et al., 2008
		Japanese encephalitis virus	5′ and 3′ UTR of genome	Anantpadma et al., 2010; Nazmi et al., 2010
		Chikungunya virus	ORF1 AUG region	Lam et al., 2015
	*Togaviridae*	Sindbis virus	5′ terminal end and AUG translation start site regions	Paessler et al., 2008
		Venezuelan equine encephalitis virus	5′ terminal end and AUG translation start site regions	Paessler et al., 2008
	*Caliciviridae*	Noroviruses	AUG region of ORF1 near the 5′ end of the genome	Bok et al., 2008
	*Hepeviridae*	Hepatitis E virus	5′ UTR and translation initiation start region of ORF1, 3′ UTR, 5′ terminus of antisense HEV RNA	Nan et al., 2015
Negative sense RNA virus	*Filoviridae*	Ebola virus	Translation initiation codon region of VP24 and VP35	Enterlein et al., 2006; Warfield et al., 2006; Iversen et al., 2012
		Marburg virus	Translation initiation codon region of VP24 and VP35	Iversen et al., 2012
	*Pneumoviridae*	Respiratory Syncytial Virus (RSV)	5′ terminus and the translation start-site region of RSV L mRNA	Lai et al., 2008
	*Paramyxoviridae*	Measles virus	Translation initiation codon region of the nucleocapsid protein mRNA	Sleeman et al., 2009
	*Orthomyxoviridae*	Influenza A virus,	Translation initiation codon regions of polymerase subunit PB1 mRNA and NP mRNA, 3′ end of NP viral genome RNA	Gabriel et al., 2008; Lupfer et al., 2008
	*Arenaviridae*	Junín virus, Tacaribe virus, Pichinde virus, lymphocytic choriomeningitis virus,	5′ termini of both genomic segments across different arenaviruses	Neuman et al., 2011


In addition to PMO targets within the 5′ terminal end of positive-sense RNA virus genomes, PMO targeting RNA secondary structures should also be considered, especially for common conserved elements among RNA viruses, such as IRES sequences. Generally, unstructured regions are more accessible to oligonucleotide binding than are structured regions, since internal structures within target RNA can impede PMO binding (Childs-Disney and Disney, 2016). However, in some cases, direct targeting of IRES sequences could inhibit replication of some viruses, including members of the order *Picornavirales* (Yuan et al., 2006; Stone et al., 2008; Tan et al., 2014). Therefore, a deep analysis of the secondary structures of target RNA may aid PMO target selection.

For PMOs evaluated for antiviral activity against negative-sense RNA viruses, current reports mainly focus on blocking translation of individual viral genes rather than direct targeting the RNA genome. For example, PMO targeting EBOV VP24 alone was sufficient to protect monkeys against lethal EBOV infection, whereas a PMOplus formulation designated AVI-7539 that targeted VP35 failed to do so (Warren et al., 2015). Therefore, screening to find effective PMO targets of negative-sense RNA viruses may require more careful consideration than needed for positive-sense RNA viruses.

Although not yet extensively studied, PMO target selection appears to be more complicated for large DNA viruses such as herpesviruses. Due to their large genome size, such DNA viruses encode many genes, including indispensable and dispensable genes. On the one hand, some indispensable genes studied so far do not appear to be good PMO targets. For example, HSV-2 early genes UL30 and UL39 encode the viral DNA polymerase and the large subunit of ribonucleotide reductase, respectively, which are essential for viral DNA replication (Eide et al., 2010). However, blocking HSV-2 UL30 and UL39 mRNA translation by PMO did not significantly reduce viral replication or transmission from infected cells (Eide et al., 2010). On the other hand, DNA viruses encode many dispensable accessory proteins with multiple functions, such as antagonist to host innate immunity. Such genes need to be carefully validated both *in vitro* and *in vivo* before use as PMO targets. For example, PMO targeting of vIRF-1 of KSHV demonstrated little protection against KSHV-infected PEL engrafts in SCID mice, in spite of its effectiveness as a PMO target *in vitro* (Zhang et al., 2011).

Aside from challenges regarding target selection, the emergence of resistant virus after sequence-specific therapy is another common challenge faced by antivirals. Indeed, a HCMV mutant with sequence-dependent resistance to the phosphorothioate oligonucleotide Fomivirsen was discovered almost simultaneously with market approval of the drug (Mulamba et al., 1998). Because PMO-based antiviral therapy is sequence-specific, PMO-resistant target mutations would abolish PMO inhibition, as has been already observed (Neuman et al., 2006). For West Nile virus, the sequencing of PPMO-resistant WNV mutants has demonstrated that viruses resistant to 5′-end PPMO treatments contained two to three mismatches within the PPMO-binding site, whereas 3′ CSI PPMO-resistant viruses accumulated mutations outside the PPMO-targeted region (Deas et al., 2007). Meanwhile, PPMO-resistant-WNV infection of mice was shown to antagonize PMO-mediated protection against virus (Deas et al., 2007). Similar PMO-resistance has been reported for Ebola virus after treatment with PMO targeting Ebola virus VP24 and VP35 as well (Kugelman et al., 2015). Therefore, mutations within or outside of PMO-targeting sequences might lead to PMO resistance and will be a future challenge.

In our research of PPMO inhibition of PRRSV, a multiple log10 reduction of viral RNA copies was achieved when PMO sequence was complementary to a conserved region within the 5′UTR of the PRRSV genome, although low level viral RNA replication was still observed. This suggests that PMO-resistant mutants might have arisen or that the effective PMO concentration at the site of viral replication was not maintained. However, since little research has been conducted to investigate genome sequences of PMO-resistant PRRSV, PMO-resistant virus mutant sequences remain uncharacterized. One possible strategy to encounter PMO-resistance mutants would be incorporating of promiscuous bases such as inosine to compensate for predicted base-pair mismatches (Warren et al., 2012). However, it is a challenge to accurately predict the potential mutation nucleotides within a PMO-targeting sequence. Therefore, the PMO target sequence against any specific RNA virus must be carefully selected to minimize generation of mutants. Moreover, use of multiple PMOs against different targets of the same virus may help to avoid mutant virus generation.

An alternative strategy to prevent PMO-resistant virus is indirect inhibition of a host factor essential for virus replication. Currently, only one of such study has been conducted on influenza virus and exploits the fact that cleavage of viral hemagglutinin (HA0) by host proteases is crucial for viral infectivity. In this study, treatment of human Calu-3 airway epithelial cells with a PPMO designed to interfere with pre-mRNA splicing of TMPRSS2 (the host protease responsible for HA0 cleavage) resulted in TMPRSS2 mRNA lacking exon 5, finally led to expression of an enzymatically inactive form of TMPRSS2 (Bottcher-Friebertshauser et al., 2011). Therefore, blocking of HA0 cleavage by this PPMO was confirmed in different human seasonal and pandemic influenza A viruses and resulted a significant reduction of viral titers (Bottcher-Friebertshauser et al., 2011). In addition to above investigation, variety host proteins have been identified as essential factors required for virus replication, host defense and viral pathogenesis in recent years (Rajsbaum et al., 2014; Tripathi et al., 2015). The putative ubiquitin ligase UBR4 was identified as a novel host factor involved in the budding of influenza virion (Tripathi et al., 2015), targeting such factor could block the release of influenza virus. Although it is unknown if UBR4 could be employed by other enveloped viruses for budding as well, it is possible that targeting a commonly used host factor which is required for viral replication could broaden the antiviral spectrum of PMO. Therefore, application of PMO to target host factor as antiviral strategy may not only avoid generation of PMO-resistant virus, but also expand the antiviral spectrum as long as the targeted host factor is employed by different virus for replication.

## Conclusion and Perspective

It has been two decades since the first approval of an antisense-based therapy. PMOs have become important ON analogs that have driven development of antisense therapies with efficacy and safety demonstrated by the recent FDA approval of Eteplirsen. Indeed, biological stability, neutral charge, and RNase H-independent mechanism of action are all unique PMO features. Moreover, besides their development as antiviral compounds, PMO evaluation as anti-cancer or anti-bacterial agents is ongoing (Enterlein et al., 2006; Warfield et al., 2006; Cansizoglu and Toprak, 2017; Chen et al., 2017; Summerton, 2017). Since PMO have a good track record as useful tools in developmental biology and as therapeutic agents, three generations of PMO have been developed (unmodified PMO, conjugated PMO, and PMOplus) to improve intracellular delivery (Daly et al., 2017). However, several issues remain unresolved before PMO are adopted for widespread use.

On the one hand, more studies are needed to establish a convenient route for PMO administration *in vivo*. In most *in vivo* studies, PMO are administrated via either intravenous or intramuscular injection. Although reports have demonstrated that PMO administered via intranasal delivery can inhibit replication of viruses with a respiratory tract tropism (Opriessnig et al., 2011; Rajsbaum, 2017), it is notable that in these *in vivo* studies employed PMOs for as antiviral agents against two respiratory viruses, influenza virus and PRRSV (Opriessnig et al., 2011; Rajsbaum, 2017). Since there is no investigation conducted to see if intravenous or intramuscular administration of PMO also effectively against influenza virus and PRRSV, it is possible intranasal delivery of PMO is preferable for virus causes respiratory infection. Moreover, it is also possible that virus-specific delivery routes based on the initial infection sites may offer a better antiviral efficiency rather than using intravenous route as universal way for PMO administration. On the other hand, although PMO is a highly adaptive platform for delivery of nucleic acid sequence-specific drugs, selection of an effective target and avoidance of the emergence of mutant virus also require more investigation. If these issues could be properly addressed, PMO should serve as a promising strategy for treatments of a variety of diseases, including difficult-to-treat viral infections. As is generally true for antiviral drug development, high efficacy, low toxicity, good pharmacokinetics, good bioavailability, and low cost are all characteristics sought in a PMO compound. Results discussed in this review show great promise and warrant further research to develop safe and effective antisense treatments for a variety of human diseases.

## Author Contributions

YN and Y-JZ designed this manuscript. YN prepared the main body of the manuscript. Y-JZ prepared the figure and revised the manuscript. All authors approved it for publication.

## Disclaimer

Mention of trade names or commercial products in this article is solely for the purpose of providing specific information and does not imply recommendation or endorsement.

## Conflict of Interest Statement

The authors declare that the research was conducted in the absence of any commercial or financial relationships that could be construed as a potential conflict of interest.
